# Intraocular Biopsy and ImmunoMolecular Pathology for “Unmasking” Intraocular Inflammatory Diseases

**DOI:** 10.3390/jcm8101733

**Published:** 2019-10-19

**Authors:** Rodolfo Mastropasqua, Emma Di Carlo, Carlo Sorrentino, Cesare Mariotti, Lyndon da Cruz

**Affiliations:** 1 Eye Clinic, Polytechnic University of Marche, 60126 Ancona, Italy; 2 Anatomic Pathology and Immuno-Oncology Unit, Center for Advanced Studies and Technology (CAST), Via L. Polacchi 11, 66100 Chieti, Italy; edicarlo@unich.it; 3 Department of Medicine and Sciences of Aging, “G. d’Annunzio” University of Chieti-Pescara, 66100 Chieti, Italy; 4 Anatomic Pathology and Immuno-Oncology Unit, Center for Advanced Studies and Technology (CAST), Via L. Polacchi 11, 66100 Chieti, Italy; carlo.sorrentino@unich.it; 5 Department of Medicine and Sciences of Aging, “G. d’Annunzio” University of Chieti-Pescara, 66100 Chieti, Italy; 6 Eye Clinic, Polytechnic University of Marche, 60126 Ancona, Italy; mariottic@libero.it; 7 Vitreoretinal Department, Moorfields Eye Hospital, 162 City Rd, Old Street, London EC1V 2PD, UK; lyndon.dacruz1@nhs.net; 8 NIHR Biomedical Research Centre for Ophthalmology, Moorfields Eye Hospital, London, EC1V 2PD, UK; 9 Institute of Ophthalmology, University College London, London EC1V 2PD, UK

**Keywords:** intraocular inflammation, intraocular biopsy, masquerade syndrome, intraocular lymphoma, endophthalmitis, molecular pathology, immunohistochemistry, laser capture microdissection, qPCR

## Abstract

Intraocular inflammation can hide a variety of eye pathologies. In 33% of cases, to obtain a correct diagnosis, investigation of the intraocular sample is necessary. The combined analyses of the intraocular biopsy, using immuno-pathology and molecular biology, point to resolve the diagnostic dilemmas in those cases where history, clinical tests, and ophthalmic and systemic examinations are inconclusive. In such situations, the teamwork between the ophthalmologist and the molecular pathologist is critically important to discriminate between autoimmune diseases, infections, and intraocular tumors, including lymphoma and metastases, especially in those clinical settings known as masquerade syndromes. This comprehensive review focuses on the diagnostic use of intraocular biopsy and highlights its potential to enhance research in the field. It describes the different surgical techniques of obtaining the biopsy, risks, and complication rates. The review is organized according to the anatomical site of the sample: I. anterior chamber containing aqueous humor, II. iris and ciliary body, III. vitreous, and IV. choroid and retina. We have excluded the literature concerning biopsy for choroidal melanoma and retinoblastoma, as this is a specialized area more relevant to ocular oncology.

## 1. Introduction

The diagnostic approach to intraocular infectious or inflammatory diseases is based on clinical history and examination supported by serologic, microbiologic, and fundamental imaging tests. The tests include ocular echography, fluorescein, and indocyanine green (ICG) angiography as well as more advanced imaging technologies. These range from computed tomography (CT), magnetic resonance imaging (MRI), and positron emission tomography (PET) to the recently introduced enhanced-depth imaging optical coherence tomography (EDI-OCT) and optical coherence tomography angiography (OCTA).

Despite the diagnostic potential of these imaging techniques, investigating the intraocular biopsy becomes essential in cases of atypical or aggressive clinical manifestations, difficulty identifying the etiology, and excluding malignancy or failure of conventional therapy because of a misdiagnosis. Currently, up to 33% of patients suffering from intraocular inflammation have undergone a biopsy [1].

The combined immunopathological and molecular diagnostics of the intraocular biopsy discriminate between infectious and non-infectious, autoimmune or neoplastic causes by using cutting-edge methods, such as laser capture microdissection (LCM) followed by polymerase chain reaction (PCR), or multiplex PCR, real-time PCR, or metagenomic deep sequencing (MDS). These procedures overcome the diagnostic limits of the traditional cytology, histology, immunohistochemistry, and flow cytometry and are suitable for specimens with few or poorly preserved cells.

The teamwork between the ophthalmic surgeon and the molecular pathologist, including planning testing of the biopsy sample, leads to a correct diagnosis and early therapy of ocular pathologies and is fundamental for discovering new molecular pathways and targets for tailored therapies.

Here, we provide a comprehensive overview of the diagnostic use of intraocular biopsy and highlight its potential to enhance research in the field. The surgical techniques of sampling and the usefulness of the intraocular biopsy will be discussed according to the anatomical sites as follows: I. anterior chamber containing aqueous humor, II. iris and ciliary body, III. vitreous, and IV. choroid and retina. The aqueous humor sampling does not involve the collection of tissue; therefore, it should not be considered a biopsy. Since in some clinical pictures the anterior chamber sampling can be diagnostic, the authors decided to describe it in this review for completeness.

## 2. Aqueous Humor Sampling

Anterior chamber (AC) paracentesis may have both therapeutic and diagnostic purposes. As a therapeutic tool, it is useful in rapidly reducing intraocular pressure (IOP) in settings such as scleral buckling, acute angle closure glaucoma, central retinal artery occlusion, and following intravitreal injections [1,2,3]. As a diagnostic technique used for determining the etiology of anterior uveitis, an AC paracentesis of approximately 100–200 μL is sufficient for both smear cytology and immunocytochemistry (to promptly identify neoplastic and inflammatory cells) as well as leaving a residual volume for further analysis, as suggested by clinical data and imaging tests [4,5,6]. Anterior uveitis (AU) implies the presence of inflammatory cells in the AC (iritis) or the AC and anterior vitreous (iridocyclitis) [7] and is found in 75% of all cases of uveitis. Clinically, using slit-lamp biomicroscopy the finding of aqueous cells is diagnostic of iritis, which is often accompanied by aqueous flare composed of albumin [8].

Anterior uveitis may occur due to a variety of causes, which entail substantial differences in the therapeutic choice, and specifically, (i) infectious agents, such as Bartonella, Borrelia Brucella, Herpes simplex 1 and 2 (HSV-1-2), Varicella zoster virus (VZV), Epstein–Barr virus (EBV), Cytomegalovirus (CMV), Toxoplasma gondii (Toxo), Leptospira, Treponema pallidum, Mycobacterium tuberculosis (TB); (ii) Immune-mediated and inflammatory (non-infectious) causes, that may be associated with systemic diseases, such as sarcoidosis, ankylosing spondylitis (HLA-B27-associated uveitis), juvenile rheumatoid arthritis, systemic lupus erythematosus, polyarteritis nodosa, Behçet disease, psoriasis, Vogt–Koyanagi–Harada disease, multiple sclerosis or inflammatory bowel diseases; (iii) neoplastic (masquerade category) and drug-induced causes related to different medications (including but not limited to rifabutin, cidofovir, zoledronate and pamidronate) and, finally, (iv) traumatic causes [9].

A large proportion of AU is non-infectious and associated with the HLA-B27 haplotype; while viral infections most commonly with those belonging to the Herpesviridae family; are regarded as an important cause of infectious AU [10].

Depending on the clinical picture the diagnostic aqueous humor (AH) tap may be analyzed by: (i) western blotting *(WB)*, to detect local antibody (Ab) production to a specific microbial pathogen; (ii) determining the Goldmann-Witmer coefficient (GWC), to quantify Ab production, (iii) enzyme-linked immunosorbent assay (ELISA) and/or by immunofluorescence assay, to detect pro- and anti-inflammatory mediators; (iv) polymerase chain reaction (PCR), to amplify and detect the DNA or RNA of specific viruses, bacteria, fungi, and parasites as well as detecting cytokine and inflammatory related transcripts and, in cases requiring differentiation from lymphomas, for assessing IgH or T-cell receptor (TCR) gene rearrangements (to identify B-cell and T-cell lymphomas, respectively) [11,12]; (v) proteome analyses with a mass spectrometer (MS) coupled to a nano-LC-2D HPLC system (ThermoFisher, Waltam Massachusetts, USA), to characterize the protein composition of AH [13]. Although further analysis and validation are required to determine its reproducibility, sensitivity, and specificity, proteomic analysis of AH is a promising strategy for diagnosing and treating a wide range of diseases of the anterior segment [13,14].

**Figure 1:**
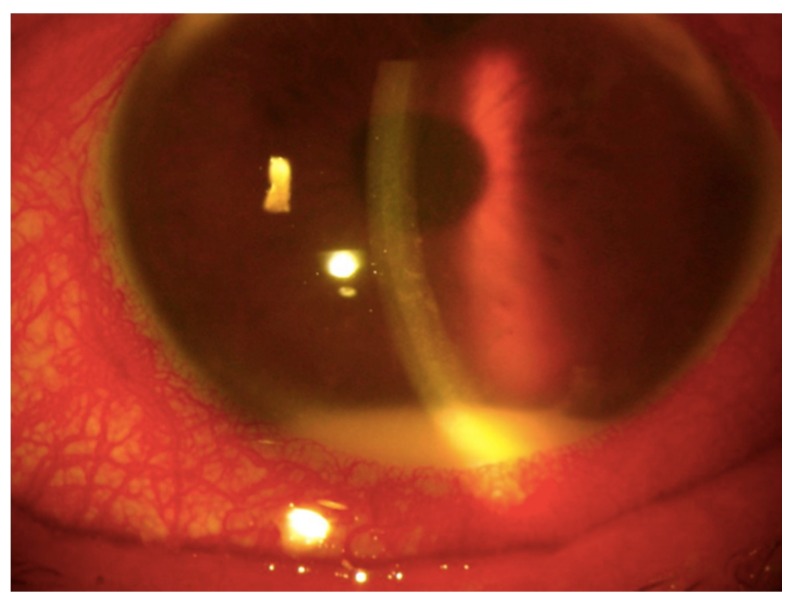
Hypopyon in a patient with infective uveitis. An anterior segment photograph of an eye of a 70-year-old male with hypopyon secondary to bacterial endophthalmitis.

Aqueous humor sampling is especially valuable diagnostically in patients with masquerade syndromes, in the presence of a hypopyon (Figure 1), a typical sign of active acute AU. The hypopyon consists of a collection of inflammatory cells, mostly granulocytes and possibly malignant lymphoid cells, in the lower angle of the AC. A herpetic etiology can be suspected in patients with iris atrophy, pigmented keratic precipitates, endotheliitis, or ocular hypertension [15,16]. Herpetic infections usually manifest as non-granulomatous uveitis in acute cases and as granulomatous uveitis in chronic cases [17,18]. Detection of the pathogens responsible for infectious uveitis, besides cytopathological examination and microbiological tests, currently relies on PCR analyses of the AH sample, which is easier to obtain than vitreous. By using a minimum volume of 0.05 mL of AH through PCR, specific DNA sequences can be amplified to provide fast identification of specific pathogens [18,19]. The GWC provides additional data to determine the pathogenic level of the virus load [10]. Combining PCR, GWC, and WB has significantly increased the diagnostic sensitivity of PCR by up to 97% [20].

Alternatively, real-time PCR, also called quantitative PCR (qPCR), provides both evidence and quantification of the load of the pathogen, and thus allows distinguishing active infection from low-grade pathogenicity. Currently, multiplex PCR allows for the amplification and detection of several different sequences at the same time. Specific genomic DNA from different viruses, bacteria, and fungi can be measured simultaneously in a small sample volume. At present, the combination of multiplex PCR and qPCR, known as a comprehensive PCR system, has further improved the diagnostics of infectious uveitis, since it combines the possibility of detecting a variety of genomic DNA (HSVs, VZV, EBV, CMV, Toxo, Parvovirus B19, BK virus, JC virus) with their subsequent quantitation. When the multiplex PCR generates positive results, then qPCR is performed to quantify the copy number of the genome in the sample and to confirm its pathogenic role [21].

Typically, AC and vitreous biopsies are performed together. In cases of acute malignant lympho-proliferative process outpacing the clearance mechanism of AC, the abundance of tumor cells within the pseudohypopyon can provide sufficient material for both cytopathology and flow cytometry (which allows for the simultaneous analysis of several different leukocyte cell surface markers) [22,23]. However, if a neoplastic pseudouveitis is suspected, the diagnostic value of AC paracentesis is limited, since malignancies or metastases primarily involve the vitreous, uvea, or retina [22,24] and the involvement of AC can be late or consisting of an inflammatory reaction, therefore vitreous or chorioretinal (CR) biopsies are preferred.

### 2.1. Research in the Field

Analyses of cytokines, chemokines, or metalloproteinases in the AH are currently being developed for diagnostic and prognostic purposes, in particular to meet the following needs: (a) differential diagnosis between primary vitreoretinal lymphoma (PVRL) and uveitis. Interleukin-6 (IL-6) is produced at high levels by inflammatory cells in uveitis, whereas IL-10 is produced by malignant B lymphocytes in intraocular and central nervous system (CNS) lymphoma [25]. PVRL is typically associated with an increased IL-10 to IL-6 ratio (greater than 1.0) [25,26,27]; (b) assessment of the risk for developing secondary glaucoma in patients with juvenile idiopathic arthritis, who have developed chronic anterior uveitis (JIAU). Increased level of TGFβ-2 in AH of these subjects has been associated with intraocular pressure elevation and occurrence of secondary glaucoma [28]; (c) monitoring the response to anti-vascular endothelial growth factor (VEGF) drug treatment for choroidal neovascularization secondary to age-related macular degeneration or pathologic myopia. Reduced levels of VEGF and pigment epithelium-derived factor (PEDF, also known as SERPINF1) in AH are associated with response to treatment and disease inactivity [29,30,31]; (d) monitoring the response to anti-VEGF drug treatment of diabetic retinopathy (DR) and diabetic macular edema (DME). Increasing inflammatory cytokines concentrations are related to more severe stages of DR [32]. Levels of VEGF, IL-6, IL-8, inducible protein-10 (IP-10), intercellular adhesion molecule 1 (ICAM-1), and monocyte chemotactic protein 1 (MCP-1) are associated with macular edema in type 2 diabetic patients [33,34]. Decreased levels of pro-angiogenic and inflammatory mediators such as IL-6, IP-10, MCP-1, PDGF-AA, and VEGF are related to a better therapeutic response and reduced impairment of foveal thickness in DME [35]; (e) monitoring the response to immunosuppressive or biological (anti-TNF-α) agents of autoimmune uveitis. The decrease in the AH levels of VEGF and IL-2 have been associated with reduced intraocular inflammation [36]; and finally, (f) the development of tailored treatment of non-infectious uveitis by targeting cytokines and their receptors. Assessment of the cytokine profile by bead-based multi-detection assays may identify molecular mediators of uveitis and provide new targets for therapy [37].

### 2.2. Surgical Technique

Several methods of AC paracentesis have been described [38,39]. Presently, AC paracentesis is usually performed using a fine gauge needle from 27 to 30 gauge (G) attached to a 1 mL syringe. AC paracentesis can be performed either under the operating microscope or at the slit lamp. Both techniques are usually carried out under topical anesthesia and aseptic conditions. At the end of the procedure, topical iodine or antibiotics should be instilled. The bevel of the needle should corneal facing to avoid iris tissue incarceration [38,39]. The use of an operating microscope allows optimal visualization of the AC during the procedure while the supine position and the surgical headrest minimize head movements. It is also possible that lens touch is reduced with the patient supine because of a slightly more posterior lens position. The use of a corneal pre-incision (partial thickness corneal incision) reduces the risk of touching the anterior capsule of the lens due to a more controlled puncture of the AC [40]. However, the advantages of an operating microscope are not significant enough to delay the AC tap especially in the presence of possible bacterial endophthalmitis.

Anterior chamber paracentesis is a safe technique with a low incidence of perioperative and postoperative complications [38,41]. The complications related to this procedure comprise trauma to the cornea, iris or lens, entry site leak, hypotony, hyphema, corneal abscess, or endophthalmitis [19,38,41]. The occurrence of hyphema was reported to be more frequent in eyes with elevated IOP [39]. In the case of higher intraocular pressure (>30 mmHg), the procedure should be preceded by the reduction of the IOP medically if possible. Some authors have advocated paracentesis in the presence of miosis to reduce lens and iris complications. However, the complication rate is already low and comparable to that reported in miosis [38,41].

Recently, Kitazawa et al. described a new technique for AC paracentesis, using a short 30 G needle combined with a disposable pipette with a squeeze bulb located at its head, which allows the aqueous humor sample to be drawn from the patient using one hand. The main advantage of this tool is to produce no dead space, allowing for the aqueous humor to be obtained efficiently, even in cases with a shallow AC. The authors reported no major complications in a series of 301 patients [42].

## 3. Iris and Ciliary Body Biopsy

Biopsies of the iris and ciliary body are primarily performed in cases of suspected neoplasia. Tumors of these structures include malignant melanoma, more rarely medulloepitheliomas, and many benign lesions, such as glioneuroma, hemangioma, leiomyomas, osteoma, and juvenile xanthogranuloma [43]. Since ophthalmic oncology goes beyond the objectives of this article, we will focus on those conditions where iris and ciliary body biopsies can help in the diagnosis of intraocular inflammation of uncertain origin. In particular, it can be useful in the investigation and management of infectious, autoimmune, and idiopathic uveitis by means of histopathology and molecular analyses. Histopathological examination of tissue can identify inflammation as acute or chronic, which is distinguished in granulomatous and nongranulomatous.

**Figure 2:**
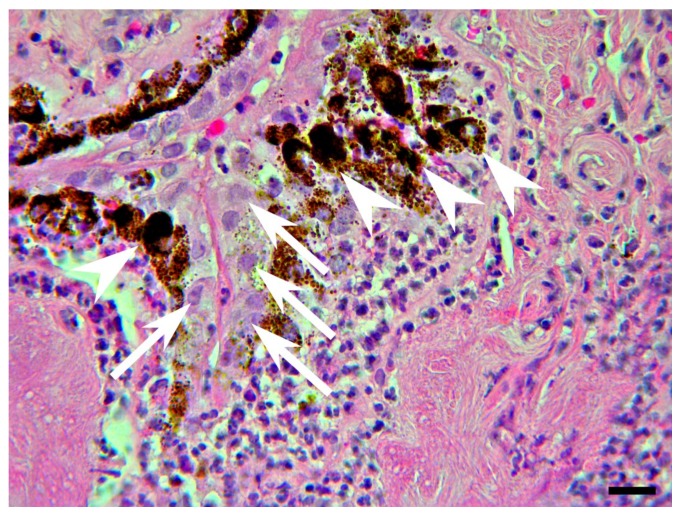
Ciliary body biopsy from a 90-years-old male with acute suppurative endophthalmitis. Hematoxylin and eosin (H&E) staining shows a massive influx of polymorphonuclear neutrophils that fragments the fibro-muscular tissue of the ciliary body and also displaces and compresses the pigmented (arrowheads) and non-pigmented (arrows) layers of the ciliary epithelium. Magnification: ×400; scale bar: 30 μm.

Acute inflammation is often infectious in origin and timely diagnosis and treatment are fundamental to optimize visual outcome. The causative organism, a bacterium (usually Gram-positive) or fungus, may complicate the ocular surgery (most frequently cataract surgery, followed by intravitreal injections, penetrating keratoplasty, trabeculectomy, and glaucoma drainage device implantation) or be introduced through a perforating wound (exogenous endophthalmitis). *Bacillus* and *Streptococcus* are common species found in penetrating trauma with an intraocular foreign body [44]. Other species isolated include Staphylococcus epidermidis, Propionibacterium acnes, Pseudomonas and Streptococcus, Gram−negative organisms, fungi, and mixed pathogens. Occasionally, the causative microorganism reaches ocular tissues through the bloodstream (endogenous endophthalmitis) and may be bacteria, mostly Streptococcus and Staphylococcus aureus in the majority of the population [45], and Klebsiella pneumoniae in East Asian people [46] as well as fungi, particularly Candida albicans and Aspergillus, and rarely parasites. The most common predisposing factors include immunosuppressive diseases, such as diabetes mellitus, HIV infection, long-term use of broad-spectrum antibiotics, steroids and other immunosuppressive drugs, indwelling intravenous catheters, and intravenous drug abuse [47,48]. Acute endophthalmitis manifests as a massive purulent reaction in the anterior and vitreous chambers, histologically represented by a massive granulocyte infiltrate, which can disarrange the fibro-muscular structure of the ciliary body, as illustrated in Figure 2. A common cause of noninfectious endophthalmitis is massive necrosis of a malignant uveal melanoma or a metastatic carcinoma [49].

**Figure 3:**
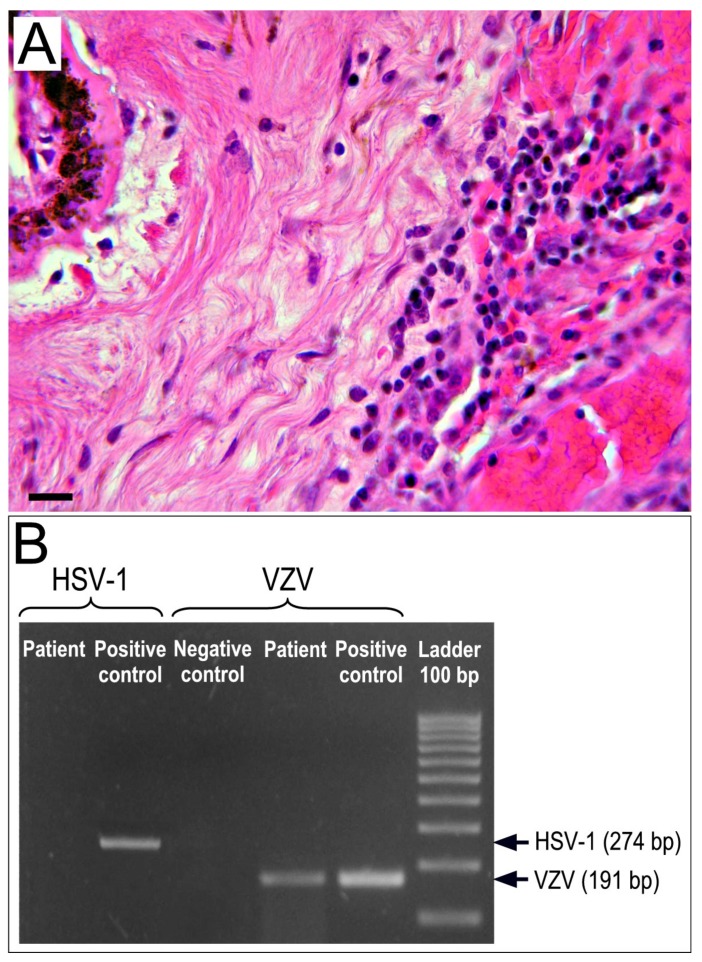
Ciliary body biopsy from a 66-year-old female with chronic non-granulomatous endophthalmitis. (**A**) H&E staining reveals lymphoplasma cellular infiltrates and micro-hemorrhagic events in the fibro-muscular structure of the ciliary body, close to the ciliary epithelium. Magnification: ×400; scale bar: 30 μm. (**B**) Polymerase chain reaction (PCR) analysis performed to detect Herpes simplex type 1 (HSV-1) and varicella zoster virus (VZV) DNA (using the following primers, HSV-1-for, 5′-CTG-CAG-ATA-CCG-CAC-CGTATT-3′; HSV-1-rev, 5′-CAT-CTT-CGA-CCG-CCA-TCCCAT-3′; VZV-for, 5′-TCC-ATC-TGT-CTT-TGT-CTTTCA-C-3′; VZV-rev, 5′-ATT-TTC-TGG-CTC-TAATCC-AAG-G-3) reveal positivity only for VZV DNA.

Chronic non-granulomatous inflammation of the eye histologically manifests as lympho-mononuclear infiltrates that primarily involve the uveal tract. In most cases the chronic inflammatory reaction has a viral etiology (primarily HSV and VZV) that can be determined by molecular tests. Figure 3 shows a lymphocytic infiltration of the iris and ciliary body (Figure 3A) and PCR detection of VZV DNA in the same tissues (Figure 3B).

Granulomatous inflammation may be the result of a specific infection such as toxoplasmosis (Toxo), tuberculosis (TB), syphilis, nematodiasis, cytomegalic inclusion, or immune system disorders such as sarcoidosis and collagen diseases [50,51]. In cases of uveitis where the etiology cannot be ascertained, the entity will be designated as idiopathic granulomatous inflammation of the uveal tract. Often, the most diagnostic lesions are not found in the iris, ciliary body, or choroid but rather in the retina, vitreous, or sclera [52].

Iris biopsy, usually combined with AC tap is required to analyze iris stromal or epithelial cysts, arising from posterior iris pigment epithelium or at the pupillary margin, associated with inflammation and/or obstructing the visual axis [43,53,54]. In such cases, histopathology is essential in the differential diagnosis between chronic granulomatous inflammations, juvenile xanthogranuloma, or malignant lesions [43,50].

Iris and ciliary body biopsy may also be diagnostic in masquerade syndromes, when lymphoma, the most common malignant orbital tumor, or metastasis, “hidden” by a reactive inflammation, are suspected [55,56,57]. Iris and ciliary body can be infiltrated by metastatic adenocarcinoma (from the breast, lung, or gastro-intestinal tract), systemic lymphoma, or by PVRL, which is rarely found here as the first site of presentation [43,52,55,56,57]. The involvement of the anterior segment manifests with abnormal iris vessels or nodules, hyphema, iridocyclitis, secondary glaucoma (including open angle, closed angle, or neovascular), or clinically visible iris/ciliary body lymphoid infiltration [56,58,59,60] and can precede detection of subretinal infiltrates [52,61].

**Figure 4:**
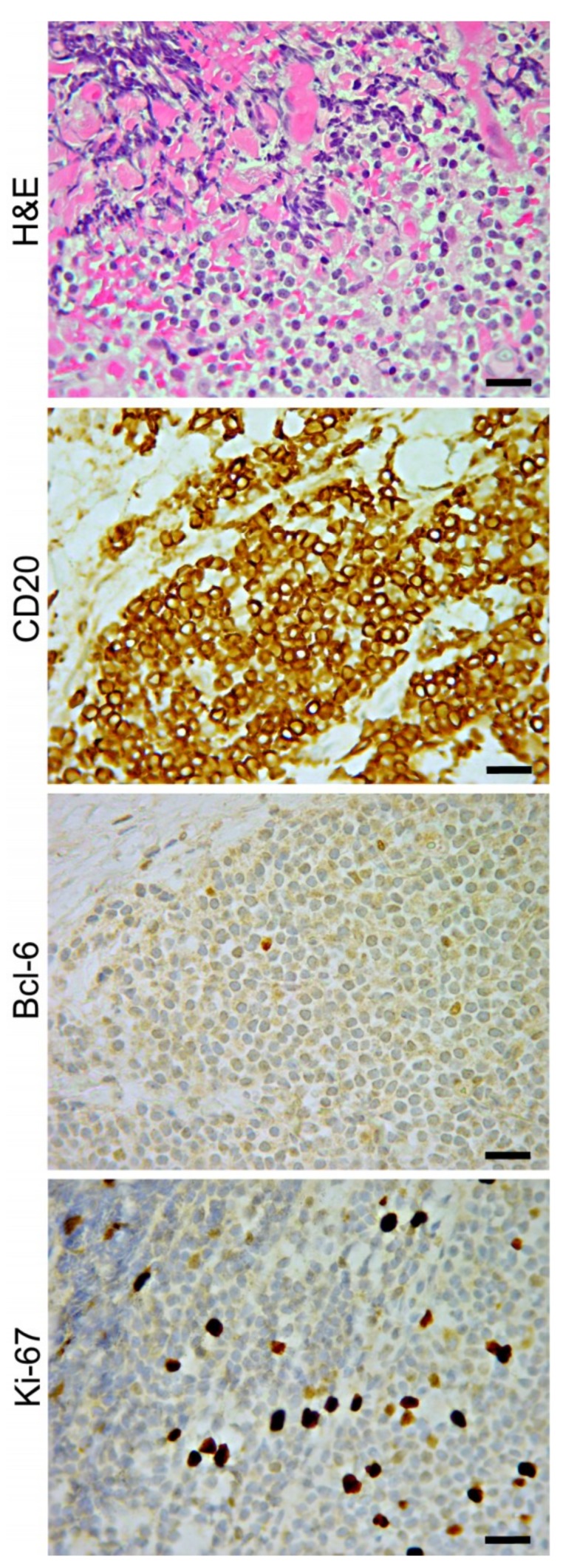
Ciliary body biopsy from an 82-year-old female with intraocular lymphoma. Extra-nodal marginal zone B-cell lymphoma of mucosa-associated lymphoid tissue (MALT) is represented by small lymphocytes with centrocyte-like features. Immunohistochemistry identifies the B cell phenotype (CD20+) and also reveals the lack of bcl-6 expression and low proliferative activity (Ki-67, 5%). Magnification: ×400; scale bars: 30 μm.

PVRL commonly affects elderly patients and manifests as a chronic uveitic masquerade syndrome that is unresponsive to corticosteroid therapy. Histologically, the majority of primary anterior uveal lymphomas are extra-nodal B-cell lymphoma of the marginal zone of mucosa-associated lymphoid tissue (MALT lymphoma) [56,62] as shown in Figure 4. Intraocular T-cell lymphomas are uncommon, and mostly represent metastasis of systemic T-cell lymphomas, including primary cutaneous peripheral T-cell lymphoma, NK-T cell lymphoma, and rarely, adult T-cell leukemia/lymphoma (ATL) [63,64], which penetrates the iris, ciliary body, and peripheral choroid.

Secondary intraocular lymphomas (SIOL) usually consists of a systemic diffuse large B cell lymphoma [59] metastasizing to the iris, ciliary body, and choroid. In comparison with PVRL, SIOL are much less prevalent; they typically manifest as a sudden and severe bilateral inflammatory reaction of the anterior segment and are less likely to create a diagnostic dilemma.

### 3.1. Research in the Field

In the last few years, in vitro and in vivo studies on the pathogenesis of ocular inflammation have revealed that receptors expressed by the iris pigment epithelium (IPE) contribute to the development of uveitis due to their ability to boost innate immune mechanisms through several inflammatory mediators produced in response to ocular pathogens. Both IPE and retinal pigment epithelium (RPE) form an interface between the eye and the environment that is not readily accessible to myeloid cells [65,66,67].

By their ability to detect signals via innate immune receptors, such as Toll-like receptors (TLRs), they can recruit myeloid cells, such as neutrophils and macrophages, to the site of injury and promote inflammation. In particular, the lipopolysaccharide (LPS) receptor complex, which consists of two interacting receptors (CD14 and TLR4) and an associated protein (MD-2) [68,69,70], when stimulated by LPS, induces the production of pro-inflammatory cytokines by IPE [71,72,73]; especially IL-8, a powerful neutrophil chemoattractant, and monocyte chemoattractant protein-1 (MCP-1/CCL2) that regulates migration and infiltration of monocytes/macrophages. Unraveling the molecular drivers of inflammation in the iris and ciliary body samples by immuno-pathological methods, PCR, WB, multiplex bead array, or biochip array technology, may in the future suggest appropriate targets for personalized treatments of uveitis.

### 3.2. Surgical Technique

Iris and ciliary body specimens are typically accessible through a corneal or limbal incision mainly with a fine needle aspiration biopsy (FNAB) [68,69,70,71]. This technique is performed under local anesthesia using the operating microscope although biopsies taken at slit lamp are described. A 22–27 G needle attached to a 10 mL syringe is required. Many authors have recommended the use of a connector between the needle and the syringe to prevent the transmission of the surgeon’s hand movements to the needle tip during aspiration [43]. Most frequently, the corneal access is either temporal or inferior, as this allows a safe planar approach to the biopsy site without interference from the eyebrow and nose. Filling the AC with a viscoelastic substance may limit possible complications. Once in the AC, the needle, with the bevel side up, is passed through the aqueous into the biopsy site. The needle should be parallel to the iris and directed in a straight line in order to avoid the pupillary margin and central visual axis. The aspiration is usually maintained over 5 s ensuring that the needle is inside the biopsy site and not merely aspirating aqueous. When the biopsy is completed, the needle has to be slowly and carefully withdrawn and the viscoelastic substance removed. Balanced salt solution (BSS) is usually used to reform the AC and normalize the intraocular pressure. In most cases, this technique does not require corneal sutures. The limit of the FNAB is related to the relatively small sample achievable and thus other transcorneal mini-invasive techniques have been described [69,71,72]. Surgical iridectomy, with or without the use of a vitrectomy cutter, has also been described as an alternative technique. Despite the larger specimen obtained, this is a more invasive procedure that often requires corneal wound sutures, with subsequent visual rehabilitation [69,73,74,75]. The iris tissue obtained, regardless of technique, is extremely fragile and should be handled with care and quickly transferred to the laboratory in an appropriate preservative.

Potential intraoperative complications are the damage of the lens and the laceration of iris blood vessels and the ciliary body causing hyphema. The occurrence of major postoperative complications related is relatively rare [76]. These comprise persistent or recurrent hyphema, prolonged hypotony, transient ocular hypertension after the AC refill, vitreous hemorrhage, and endophthalmitis [43,69].

## 4. Vitreous Biopsy

The vitreous plays a crucial role in the pathogenesis of many ocular inflammatory diseases and it is also the compartment where cells and inflammatory mediators are released coming from pathologic processes involving the choroid, retina, optic nerve, or ciliary body [77]. Vitreous sampling can be obtained by pars plana (three-port) vitrectomy (PPV) or FNAB [26,60,78,79]. It is usually performed as the primary procedure, when an invasive investigation is necessary because (a) it is more likely to lead to a diagnosis than the AC paracentesis [80] and (b) it has a lower risk of complications than the CR biopsy [81]. In clinical practice, the primary use of vitreous biopsy has been to identify the causes of exogenous or endogenous endophthalmitis [82,83,84,85].

Specific indications for vitreous biopsy include: (1) providing a diagnosis in patients with posterior uveitis when the etiology remains uncertain after clinical examination and ancillary tests. An example is a PVRL, which manifests as persistent uveitis and requires pathological confirmation to start the treatment [86]; (2) investigating failure of conventional therapy, which may be due to a misdiagnosis, resulting in intractable disease; (3) diagnosing a sight-threatening disease that necessitates an invasive approach for diagnosis and, at times, also for treatment, such as in the cases of infectious endophthalmitis [87] and acute retinal necrosis (ARN) [88].

To reach the correct diagnosis in the cases of inflammatory, infectious, or neoplastic diseases of uncertain origin, the vitreous biopsy can be examined by a variety of laboratory tests, such as culture and microbiological tests, histopathology (for PPV) or cytology and immunocytochemistry (for FNAB), PCR methods, flow cytometry, Ab measurement (by GWC), and cytokine evaluation (by ELISA) [86]. The sample is processed based on clinical features and the suspected disease, taking care to proceed rapidly to avoid necrosis of the few inflammatory or neoplastic cells recovered.

The biopsy specimen should be divided into three samples: the first to be fixed for routine histopathological evaluation; the second to be frozen in optimal cutting temperature (OCT), the embedding compound for immunopathological and molecular characterization; and the third intended for the culture of bacteria and other microorganisms. If the biopsy specimen is not sufficient for all of these processes, it should be processed for frozen sections to allow histopathology, immunohistochemistry, and molecular analysis.

Histologic or cytologic examinations of vitreous samples are generally the first step in the pathologic diagnosis of PVRL, which in this site usually arranges as neoplastic sheets [26,52,60,89,90,91,92,93,94]. Recently, it has been demonstrated that cell block preparation is more useful for diagnosis of vitreoretinal lymphoma than conventional smear cytology [95]. This method, which employs the retrieval of small tissue fragments from a cytologic specimen that are processed to form a paraffin block, increases cellular yield and obtains numerous tissue sections for multiple immunostainings [96].

Flow cytometry and immunohistochemistry allow the identification of lymphoma cells and demonstrate their monoclonality by B-cell and plasma cell markers (CD19, CD20, CD22, BCL-6, MUM1) and kappa or lambda light chain restriction [94].

Assessment of cytokine production, specifically IL-6 and IL-10, discriminates between PVRL and other ocular inflammatory processes. As in the AH, an association of PVRL with an increased IL-10 to IL-6 ratio has also been found in the vitreous [25,26,27,97].

Molecular analysis of a vitreous specimen using PCR techniques has two main indications: (1) to diagnose PVRL and (2) to detect and identify the DNA of specific microorganisms in cases of infectious uveitis [98]. For PVRL diagnosis, PCR is used to detect monoclonality within the variable region of the third complementary determining region (CDR3) in the immunoglobulin heavy chain gene of malignant B cells. PCR and microdissection have also been used for the detection of the t(14:18) translocation (i.e., translocation between the genes for BCL-2 and IgH) [99,100].

The ageing population and the increase in patients with immunodeficiency (including HIV infection) or immunosuppression have dramatically increased the incidence of vitreoretinal lymphoma (VRL). The disease is inevitably accompanied by an inflammatory reaction and often masquerades as intraocular inflammation [101]. PVRL is a malignant non-Hodgkin’s lymphoma (NHL) which either occurs independently to, or in association with, primary central nervous system lymphoma (PCNSL). It involves the retina, the vitreous chamber and/or the optic nerve (vitreoretinal form) and has to be distinguished from secondary intraocular lymphoma (SIOL), an ocular manifestation of systemic NHL, which predominantly infiltrates the uveal tract, particularly the choroid (uveal form) [60,102]. Most PVRL are diffuse large B-cell lymphomas (DLBCL), according to the updated World Health Organization (WHO) lymphoma classification [103]; SIOL usually corresponds to the subtype of systemic lymphoma [60]. Intraocular T-cell lymphomas are rare, and most cases occur secondary to primary cutaneous or other systemic T-cell lymphomas.

In order to improve the diagnostic yield, PPV is the procedure of choice and may be both diagnostic and therapeutic, particularly in patients with significant vitreous opacity [86,104]. Mudhar and Sheard underlined that a full PPV may be preferred to a core vitrectomy to reduce the rate of false negative results [105]. They claim that inflammatory and lymphoma cells, mostly located in the cortical vitreous, have less chance to be sampled if a single core vitreous biopsy is performed.

In terms of a demonstration of the combination therapeutic and diagnostic effect of vitrectomy, the randomized multicenter clinical trial known as “The Endophthalmitis Vitrectomy Study (EVS)”, has demonstrated that immediate PPV is of substantial benefit for the treatment of postoperative bacterial endophthalmitis in patients with visual acuity of light perception (LP) [85]. It gives a chance of visual improvement due to the removal of the vitreal opacity and provides a very large volume sample for diagnostic purposes.

### 4.1. Research in the Field

As the vitreous contains proteins, proteoglycans and small molecules that originate from within and outside the eyes, and that change qualitatively and quantitatively following vitreoretinal diseases, the vitreous proteome is being studied to determine the molecular mechanisms of these pathologies. The advent of mass spectrometry (MS)-based proteomic technologies (i.e., matrix-assisted laser desorption/ionization time of flight (MALDI-TOF) mass spectrometry and liquid chromatography-tandem mass spectrometry (LC-MS/MS)) have facilitated de novo identification and quantification of a large number of proteins within a relatively small sample. In particular, the proteomic analysis of vitreous has provided novel insights into the etiology of diabetic retinopathy and candidate targets for treatments, while it looks promising to identify factors predicting risk and functional outcomes of interventional therapies [106,107].

### 4.2. Surgical Technique

Vitreous biopsy can be obtained under local anesthesia, by mean of FNAB (topical or subconjunctival infiltration) or PPV [108]. In the FNAB technique, a short 27 or 25 G needle on a syringe is inserted 3.5 to 4 mm away from the limbus (depending on lens status) and vitreous fluid is aspirated. If the first aspiration does not yield fluid, the needle can be withdrawn slightly, and aspiration may be attempted again. It is important to avoid the crystalline lens, as well as the retina when entering the eye with the needle. This technique is considered relatively safe [109] although major complications such as hemorrhage, retinal detachment, proliferative vitreous retinopathy, and endophthalmitis have been described [78,109,110].

**Figure 5:**
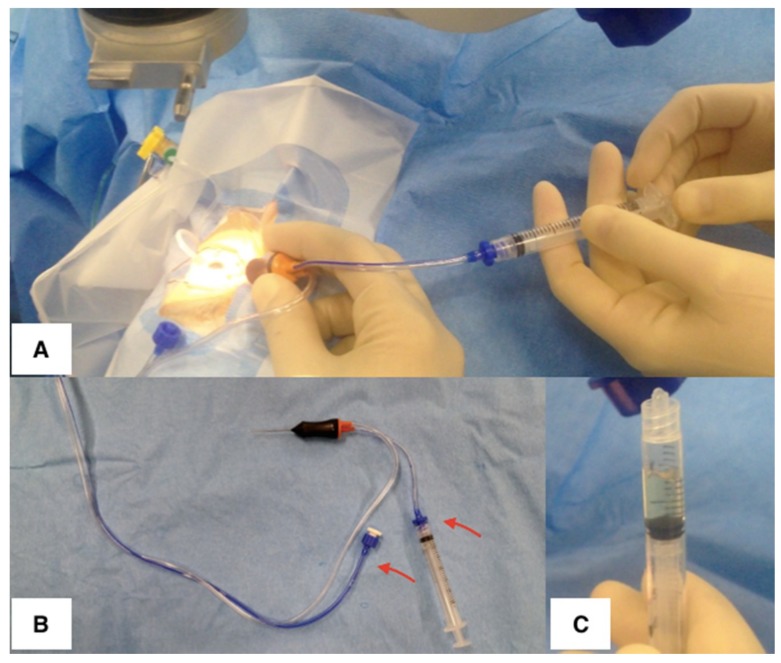
Obtaining a vitreous sample with vitrectomy. (**A**) The assistant is manually aspirating via a syringe while the surgeon is cutting with the vitrector. (**B**) The vitreous cutter shows the broken aspiration tube (arrows) connected directly to a 2 mL syringe used to obtain a biopsy. (**C**) Undiluted vitreous specimen obtained using the vitreous cutter.

Biopsies with PPV have been routinely described with 20, 23, or 25 G equipment with a move to 27 G being reported more recently [111]. The procedure can be limited to a diagnostic core vitrectomy or can be extended to a full vitrectomy with an induced posterior vitreous separation (PVD) that provides the opportunity to achieve both a diluted and an undiluted sample. In most cases, an infusion port is placed but not turned on before the ‘neat’ biopsy has been completed to avoid diluting the sample. One or two further sclerotomies are placed in the superonasal and superotemporal quadrants. A two-port sample can be taken if only a core sample is desired and the search for retinal tears can be carried out with the indirect ophthalmoscope. The neat sample is taken with manual aspiration into a syringe through the broken aspiration line while the vitrectomy cutter is activated (Figure 5A). An assistant applies gentle suction using the syringe to aspirate the neat core vitreous sample (Figure 5B). It is possible to obtain 2 mL of vitreous this way, but it is more common to aspirate 0.5–1.0 mL (Figure 5C). The surgeon maintains the IOP by external pressure on the globe with a squint hook (or other instruments) to counter the loss of intraocular volume. The vitrectomy cutter is withdrawn while still ‘cutting’ and the sclerotomies are closed as usual.

The advantage of a formal vitrectomy over a vitreous tap is the volume achieved and the large undiluted specimen allows extensive laboratory analyses [105]. Any cytokine analysis or diagnostic testing measuring specific concentrations (e.g., cytokine levels, qPCR testing) should be performed using undiluted vitreous because a specimen diluted by infusion fluid renders the concentrations inaccurate and falsely low. The diluted sample and vitreous cortical cells, contained in the vitrectomy cassette, can be used for cytopathology, flow cytometry, or other laboratory tests, after being centrifuged to concentrate the cells [86]. Since cells removed from the vitreous are liable to rapid degeneration unless fixed or exposed to the tissue culture medium promptly [75,112], the vitreous specimen has to be transported quickly for laboratory analysis. Although diagnostic vitrectomy has been considered a relatively safe procedure, the occurrence of complications such as intra and post-operative vitreous hemorrhage and retinal detachment (most commonly) as well as choroidal effusion, proliferative vitreoretinopathy, or endophthalmitis are frequently reported [84,113,114,115]. Extension of the lymphoma through the sclerotomy port to the epibulbar space following vitrectomy has also been described as a rare complication of a vitrectomy-based biopsy [116].

## 5. Chorioretinal Biopsy

The limited number of publications on CR biopsies reflect the rarity of its use due to the complexity of the surgical procedure, the risk of serious complications, and the fact that the diagnosis is frequently reached with more straightforward and less invasive techniques. Indications for a CR biopsy primarily includes the suspicion of malignancy, progressive sight-threatening retinal or choroidal lesions (retinitis and choroiditis) unresponsive to therapy, sight-threatening involvement of the second eye despite treatment, and negative vitreous analysis after multiple diagnostic biopsies and vitrectomies [117,118]. Mastropasqua et al. proposed the level of vitritis as an index of likelihood in achieving a definitive diagnosis by means of a CR biopsy in masquerade syndromes [119].

When the sensory retina, RPE, or choroid are the only ocular tissues involved in the disease, the choice of proceeding directly with a CR biopsy and avoiding a diagnostic vitrectomy, can prevent the risks linked to multiple surgeries [120,121,122]. The surgical technique for performing a CR sampling can either be FNAB, full-thickness CR biopsy or trans-scleral biopsy using a partial scleral thickness flap, depending on the location of the lesion [81,108,123,124].

Obtaining CR specimens is effective in the diagnosis of infectious retinitis, such as herpes simplex virus, caricella zoster virus, Cytomegalovirus, Candida albicans, Pneumocystis jirovecii (previously known as Pneumocystis carinii), Toxoplasma gondii and Mycobacterial [81,118,125,126,127,128,129]. However, a CR biopsy is not the primary diagnostic approach for these diseases, since analyses of intraocular fluids (aqueous or vitreous to detect microbial DNA by PCR or to quantify specific Abs), blood, or cerebrospinal fluid associated with imaging testing (optical coherence tomography (OCT) and fluorescent or indocyanine green angiography) are usually performed first and sometimes prove conclusive [130,131].

Handling of the CR biopsy depends on the amount of tissue harvested. To avoid necrosis, the ocular tissue must be rapidly divided (in a sterile manner under a dissecting microscope) and included in selected tissue culture for microbiology, fixed in formalin for histopathology, fixed in glutaraldehyde for electron microscopy (assessment of viral particles), or frozen for molecular biology. Consultation of the molecular pathologist will be decisive in choosing the most appropriate test.

**Figure 6:**
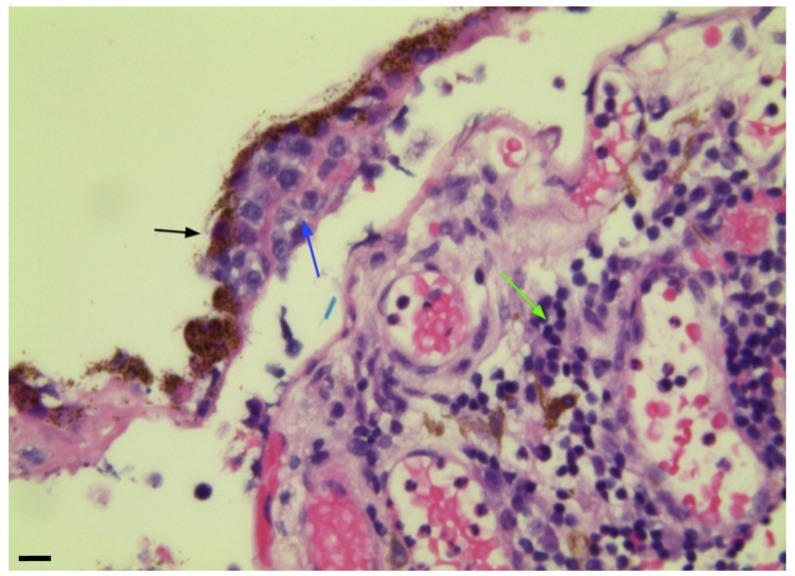
Primary vitreoretinal lymphoma in a 68-year-old female. Pleomorphic lymphomatous cells (blue arrow), lying between the retinal pigment epithelium (RPE) (black arrow) and the underlying choroid. The choroid has a reactive infiltrate of small non-neoplastic lymphocytes (green arrow). H&E staining used; magnification: ×400; scale bar: 30 μm (Department of Eye Pathology, UCL Institute of Ophthalmology 2014).

The analysis of CR samples is of paramount importance in the diagnosis/exclusion of malignancy, particularly in lymphoma [60,75,81,119,121,132,133,134,135,136]. Primary vitreoretinal lymphoma (PVRL) is the most common intraocular lymphoma [137]; usually, a high-grade diffuse large B-cell malignancy manifests with choroidal infiltration, vitritis, or even necrotizing retinitis are unresponsive to anti-viral, anti-bacterial, and anti-parasitic therapy. In PVRL, the main site of involvement is the sub-RPE space (between RPE and Bruch membrane) [124] since lymphoma cells express specific ligands, leading to preferential homing to the RPE (Figure 6); therefore, the diagnostic rate of CR biopsy is high. When an internal approach is used specimens must be taken from the deeper part of the lesion, near the choriocapillaris, where viable lymphoma cells are most likely to be found since sub-retinal tumors can be largely necrotic [59,124].

**Figure 7:**
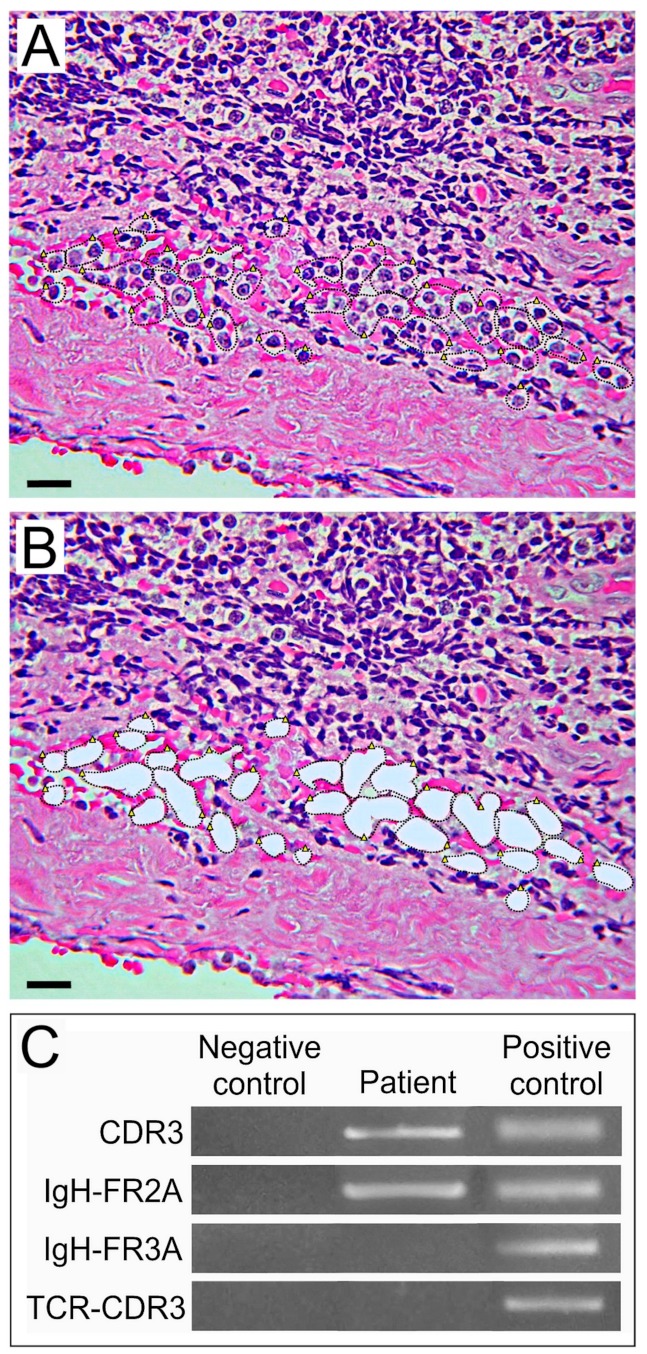
Laser capture microdissection (LCM) of lymphomatous B cells performed on a chorioretinal biopsy from an 85-year-old female with intraocular lymphoma. (**A**) H&E staining shows few lymphomatous cells confined close to the sclera by a significant chronic inflammatory reaction. (**B**) The lymphomatous cells were dissected and removed from the tissue section by LCM. Magnification: ×400; scale bar: 30 μm. (**C**) PCR analysis performed to assess the B or T cell origin of the lymphomatous cells (using three primer pairs: CDR3, IgH-FR2A, and IgH-FR3A, specific for IgH gene rearrangements and one primer pair, T-cell receptor (TCR)-CDR3, specific for TCR gene rearrangements) reveals positivity for IgH-FR2A and IgH-CDR3 and the lack of TCR rearrangements, thus identifying the B cell origin of the cancer cells.

In most cases, lymphoma cells are interspersed with reactive lymphocytes and necrotic cell debris, thus reducing the purity of DNA for molecular analyses and making the results unreliable. Laser capture microdissection (LCM) by isolating lymphoid cells from tissue sections, may increase the sensitivity and specificity of PCR from an average of 60% to nearly 100% [11]. In the case illustrated in Figure 7, atypical lymphoid cells were isolated by LCM, from a paraffin-embedded intraocular tissue sample and, after DNA extraction, PCR was performed by using the specific primers IgH-FR2A, IgH-FR3A, and IgH-CDR3 for immunoglobulin IgH rearrangements, and a specific primer TCR-CDR3 for TCR rearrangements. The positivity for IgH-FR2A and IgH-CDR3 and the lack of TCR rearrangements identify the B cell origin of the microdissected lymphoma cells.

### 5.1. Research in the Field

The use of LCM followed by PCR, transcriptomic or proteomic analyses, has proven to be effective for the diagnosis and/or molecular and genetic investigations of vitreous biopsy samples with few or poorly preserved cells. Very promising is the application of untargeted metagenomic deep sequencing (MDS) to the clinical diagnosis of infectious diseases. This revolutionary high-throughput sequencing technology overcomes limitations of traditional methods based on targeted approaches to specified infectious agents (leading to potential missed pathogens) or specific genetic aberration (leading to overlook of less common cancer driver mutations). MDS allows for an unbiased and rapid method for detection of all pathogens (fungi, parasites, DNA and RNA viruses) [138] in a small volume specimen and simultaneously, provides genetic information (chromosomal translocations, common and rare mutations, gene rearrangements) for the diagnosis of non-infectious intraocular diseases, such as malignant lymphoproliferative disorders [139].

### 5.2. Surgical Technique

Chorioretinal biopsy has been described, both under general or local anesthesia, and through both external (transscleral) or internal approaches (transvitreal or FNAB).

The transscleral approach was the first to be described but is currently rarely performed due to the risk of suprachoroidal hemorrhage and other serious complications [140,141]. For lesions anterior to the equator and especially those around the ora, however, an external approach is sometimes more practical for access. The intraocular approach provides advantages of direct visualization of the lesion and gives visualized control of intraocular bleeding with internal tamponade, and diathermy if needed [86].

The external technique requires a conjunctival peritomy and the isolation of the rectus muscles. The biopsy site is marked on the sclera, and a nearly full-thickness scleral flap (generally 6 × 6 mm) is performed. Thereafter, the flap is retracted, and diathermy is applied to the outer margin of the choroidal bed. A sharp blade is then used to incise the choroid, and the biopsy specimen is grasped with forceps. Vannas scissors may then be used to excise the specimen. The biopsy specimen is placed in fixative. The scleral flap is then sutured back into place with 7-O Vicryl sutures [118].

**Figure 8:**
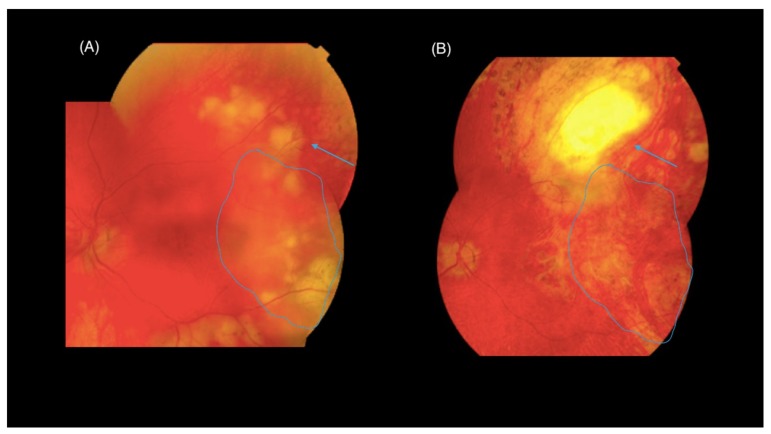
Chorioretinal biopsy. (**A**) A fundus photograph of the left eye of a 68-year-old patient with diffuse yellowish infiltrate suspected of having vitreoretinal lymphoma. (**B**) A fundus photograph of the same eye filled with silicone oil, three months after a chorioretinal biopsy. The specimen was diagnostic for “large B-cell lymphoma”. Note the biopsy site (arrows) and the regression of the tumor after a cycle of chemotherapy (encircled by dotted line).

The transvitreal approach that was described by Freeman [142] and further developed in several variations [117,118,128,133] is considered safer and more reproducible [119,125,133]. In this technique, the biopsy site should be chosen preoperatively and should include the junction of the involved and uninvolved retina, as the margins of the lesion have a higher probability of showing the pathologic process [118] (Figure 8). Central areas may have only necrotic tissue present providing inconclusive histology [142]. At the beginning of the procedure a neat vitreous sample is often obtained as described before. A complete standard PPV is then performed. In the biopsy area, retinal vessels must be treated with endocautery to limit subsequent bleeding. The choroidal area to be cut is then delineated with a diode laser or full-thickness diathermy. The retina and choroid are cut through to bare sclera using vertical cutting segmentation-scissors. Soft-tipped cannula or forceps are then used to remove the specimen through an enlarged sclerotomy, especially if the specimen is particularly large. The biopsy site is encircled with endolaser followed by tamponade with long-acting gas or silicone oil. Modern surgical techniques have dramatically decreased the incidence of complications from all vitreoretinal procedures including CR biopsy, but it remains the most high-risk intraocular biopsy technique available [133]. Reported complications of this surgery include cataract, retinal detachment, vitreous hemorrhage, choroidal hemorrhage, proliferative vitreoretinopathy, and endophthalmitis [73,81,117,119,123,128,133,143].

The FNAB for chorioretinal lesions is performed using a 25 to 30 G needle connected via a 12–18 in. segment of plastic tubing to a 10-mL aspirating syringe [68,118]. Connector tubing is used so that there will be no induced movement of the needle as the assistant exerts suction in the line for aspiration. The needle is inserted via the pars plana under direct visualization with either the operating microscope or the indirect ophthalmoscope, and the tip of the needle is advanced until it is within the lesion. Aspiration is performed multiple times. Then the needle is carefully withdrawn from the eye. Although the potential intra-operative risks are the same of the transvitreal procedure, the FNAB technique seems to be less invasive and therefore, has and causes fewer complications relative to CR biopsy performed by the internal approach [122,133,143].

## 6. Conclusions

Intraocular biopsy is essential for a correct diagnosis in many cases of ocular inflammation. A significant number of these conditions manifest a persistent and/or progressive disease, which can result in severe visual impairment due to damage to the ocular structures. Timely and definitive diagnosis and subsequent appropriate and specific therapy may be sight saving. As such, the most common indication for intraocular biopsies is atypical uveitis, when a diagnosis of malignancy or infection may be suspected, or chronic and non-resolving uveitis. Ocular lymphoma remains the common malignancy that is diagnosed with ocular biopsy and in these situations, a definitive diagnosis is central to reducing morbidity and mortality. Modern vitrectomy techniques with endoresection, as well as more reliable vitreous and aqueous sampling, obtain appropriate tissue samples more consistently, with a relatively low risk of complications. These biopsy techniques, together with cutting-edge molecular tools, enable both the achievement a definitive diagnosis and the development of tailored therapies for inflammatory eye diseases.

## 7. Method of Literature Search

PubMed and Medline advanced searches were undertaken. Only English language articles from peer review journals were evaluated. A total of 372 articles were retrieved, of which 143 articles were relevant and were included in this review.
